# 8.G. Workshop: European Health Data Space: Dialogue on TEHDAS JA data quality & semantic interoperability proposals

**DOI:** 10.1093/eurpub/ckac129.497

**Published:** 2022-10-25

**Authors:** Organised by: TEHDAS JA WP6, Chair persons: Enrique Bernal-Delgado (Spain), Persephone Doupi (Finland)

## Abstract

The EU Data Strategy foresees the development of shared European data infrastructures, enabling cross-border exchange and availability of comparable and high-quality data. The European Health Data Space (EHDS) is the first of these infrastructures to be launched, making use of the potential of health and social data for the purpose of advancing public health, research and innovation. Quality of digital health data has been the focus of research and debate for a long time and several proposals for improvement have been put forward. While these efforts are ongoing, secondary use or re-use of health data, combined with recent developments in data analytics, AI and real-world data (RWD), have raised new requirements on our understanding of data quality and the means for its assurance. Both the European Commission and Member States have underlined the need to support EU and national authorities, as well as the scientific community to agree on interoperability guidelines addressing data quality and semantic interoperability. The TEHDAS Joint Action has responded to these expectations through work undertaken in the context of WP6 - Excellence in data quality. With expected delivery deadline early 2023, the EUPHA conference offers an excellent platform to present the draft forthcoming proposals to the public health community and engage in meaningful dialogue with participants. Key members of the WP6 team will present the process through which the TEHDAS JA recommendations on data quality and semantic interoperability have been developed, the results achieved thus far and the remaining open questions. The workshop discussion section will allow the JA team to collect direct input and feedback for utilization in the production of the final version of the deliverables.

**Key messages:**

• Increased awareness and understanding of background and rational of forthcoming proposals on data quality and semantic interoperability in the European Health Data Space.

• Opportunity to raise concerns and proposals for improvement and thus have a direct impact on the final TEHDAS JA output on data quality.

